# The evolving nexus of women’s empowerment and child nutrition in India

**DOI:** 10.3389/fpubh.2025.1583678

**Published:** 2025-08-26

**Authors:** Bharti Singh, Shri K. Singh

**Affiliations:** Department of Survey Research and Data Analytics, International Institute for Population Sciences, Mumbai, India

**Keywords:** undernutrition, child nutrition, women’s empowerment, socioeconomic, NFHS, India

## Abstract

**Background:**

India has one of the highest burdens of child undernutrition, globally. Undernutrition is a persistent challenge despite the country’s economic growth. Empowering women is essential in addressing child undernutrition since empowered mothers are more inclined to obtain healthcare, enhance dietary diversity, and make informed choices that benefit their children’s health outcomes.

**Objective:**

The study aims to investigate the relationship between women’s empowerment and child undernutrition in India from 2006 to 2021.

**Methods:**

This study is based on three recent rounds of the National Family Health Survey. A composite index of women’s empowerment has been used to measure women’s empowerment. Further, binary logistic regression and decomposition analysis have been used to analyze the association and identify the key determinants that contribute to the reduction of undernutrition among children in India.

**Result:**

Our research offers significant insights into the evolving dynamics of child undernutrition in India, particularly concerning the linkages with women’s empowerment. While empowerment was statistically insignificant in NFHS-3, it became a significant driver of child undernutrition in NFHS-4 (−0.12**[−0.21, −0.03]) and NFHS-5 (−0.15***[−0.24, −0.06]). Additionally, birth order, birth weight, and mother’s BMI are critical determinants of undernourishment status among children under age five. Wealth remains a consistently significant factor across all three survey rounds. Decomposition analysis further reinforces the significance of women’s empowerment, demonstrating that it accounts for a 3.3% reduction in child undernutrition In India.

**Conclusion:**

The study underscores the critical role that empowering woman plays in combating child undernutrition, indicating the need for comprehensive strategies that prioritize women’s empowerment alongside other critical determinants to effectively tackle the persistent challenge of child undernutrition in India.

## 1 Introduction

*In the tapestry of progress, the empowerment of women weaves the threads of a nourished generation and a brighter future for India.*

Child malnutrition is a multifaceted issue that transcends geographical boundaries. Malnutrition is a deficiency, excess or imbalance in an individual’s intake of energy and/or nutrients. The consequences of malnutrition are dire as it not only stunts physical growth but also leads to a spectrum of morbidities and child mortality (48). The urgency of addressing this issue cannot be overstated, as malnourishment among children extends to their adulthood, restricts a person’s life biologically and diminishes the quality of their life (1, 2).

In 2022, Globally, 149 million (22.3%) children under age five were stunted, 45 million (6.8%) wasted, and 37 million (5.6%) were overweight or obese (3). Undernutrition is prevalent in developing regions, reflecting the complex relationship among socioeconomic disparities, inadequate healthcare, and insufficient access to nutritious food (2, 4, 5). Despite economic progress and advancements in healthcare, child malnutrition remains a formidable challenge for India. The recent round of the National Family Health Survey reveals a stagnant scenario. While there have been marginal improvements from 2005–06 to 2019–21 in stunting (decline of 12 percent point), wasting (decline of 1 percent point), and underweight (decline of 11 percent point), the rates continue to be frightening (6).

There are three key domains are used to comprehend malnutrition among children: stunting, wasting, and being overweight. Low height for age, or stunting, is a sign of chronic undernutrition caused by inadequate nutrition over a long period, including recurrent and chronic illnesses (7). Children who were stunted in their early childhood reported poor psychological functioning in their youth and suffered from higher levels of anxiety, depressive symptoms, and low self-esteem (2). In contrast to stunting, wasting reflects acute undernutrition, yet it has an equally profound impact. It has an immediate effect on the child’s health, leading to weight loss, development, and susceptibility to infectious diseases (8). Alongside these, underweight, defined as low weight for age, captures both chronic (stunting) and acute (wasting) forms of undernutrition. Underweight children face elevated risks of delayed cognitive development, poor school performance, and higher mortality, underscoring its role as a critical marker of child well-being (8). Overweight, the opposite of wasting, indicates overnutrition (8).

Numerous studies have explored the association between different socioeconomic factors and child nutrition, shedding light on the vivid nature of this challenge (9–12). Studies have revealed that undernutrition is more prevalent among children of lower birth weight and higher birth order under the age of five (11, 13). Maternal socioeconomic status emerges as a robust predictor of child undernutrition in India, suggesting that addressing multi-generational poverty and improving environmental factors are promising investments (10). Along with socioeconomic conditions, parental nutritional status, dietary diversity, hygiene practices, and women’s empowerment significantly contribute to child nutrition (9, 14). Some studies have independently linked women’s empowerment to improved childhood nutritional status, advocating for comprehensive interventions that integrate strategies for women’s empowerment (15, 16). Women play a pivotal role in shaping child nutrition as primary caregivers. Empowered women are better equipped to make informed decisions about nutrition, healthcare, and sanitation, thus positively influencing the nutritional outcomes of their children. Evidence from regions like Maharashtra and marginalized communities in Karnataka reaffirms the pivotal role of women’s empowerment in improving child nutrition, showcasing significant declines in child nutrition linked to women’s empowerment initiatives (17, 18). However, despite this recognition, only few studies have comprehensively explored how women’s empowerment impacts child nutrition over time, especially in the context of India.

Initially, scholars defined women’s empowerment as the ability to make choices for themselves and their families, emphasizing access and control over marital and social resources within families, communities, and society (19). This definition evolved over time and included the ability to influence and control one’s environment (20). Researchers expanded the concept to encompass control over resources, participation in economic decisions, self-esteem, mobility, and freedom from domestic violence, highlighting empowerment as a multidimensional construct (21–25). However, India’s contextual reality adds complexity, as some regions still struggle with conservative beliefs, indicating the long journey ahead to achieve equality (21).

India has implemented various policy interventions to improve women’s empowerment and maternal and child health. Despite these efforts, nearly one-third of India’s children are undernourished, highlighting the critical public health challenge. Furthermore, there remains a significant gap in understanding how the relationship between women’s empowerment and child undernutrition has evolved over time in India. Although existing studies have emphasized the importance of women’s empowerment, they often face challenges in encompassing the multiple dimensions necessary for a comprehensive understanding of this complex concept. To address these gaps, our study aims to investigate the relationship between women’s empowerment and their child nutrition outcome in India using data from 2006 to 2021.

## 2 Methods and materials

### 2.1 Data

The study is based on secondary dataset. The study used data from the three most recent rounds of the National Family Health Survey (6, 26, 27). The NFHS is a major, nationwide, large-scale, and multi-round survey conducted in a representative sample of households at the national, state and (from 2015 to 16 onwards) at district levels. The NFHS is an Indian version of the Demographic and Health Survey (DHS) that provides consistent and reliable estimates of fertility, mortality, family planning, child nutritional status, morbidity, utilization of maternal and child health care services, anemia, utilization and quality of health and family planning services. NFHS-3 collected information from 109,041 households, 124,385 women aged 15–49. NFHS-4 covered 699,686 women from over 601,000 households across 640 districts. NFHS-5 fieldwork for India was conducted in two phases, phase one from 17 June 2019 to 30 January 2020 and phase two from 2 January 2020 to 30 April 2021, extended due to the COVID-19 pandemic. NFHS-5 has gathered information from 636,699 households, 724,115 women aged 15–49.

### 2.2 Target population

We have taken children under the age of five who live with their mother (aged 15–49 years). The mother should be currently married or living in a union, and not pregnant.

### 2.3 Variable description

#### 2.3.1 Outcome variables

We have used underweight as the indicator of undernutrition among children under age five. Underweight is defined as a weight-for-age of 2 standard deviations (SD) or more below the corresponding median of the reference population.

#### 2.3.2 Predictor variables

Women’s empowerment is created using Confirmatory Factor Analysis (28, 29). We have taken 27 items to construct the index under six domains named attitude toward violence, decision making, perceived sexual rights, freedom of mobility, financial security and societal norms. The description of each domain is in the Supplementary 1.

The result of the goodness of fit indices shows that the RMSEA value in all survey years was below the cutoff point, which was 0.05. The CFI and TLI values were higher than 0.950, indicating strong reliability. Finally, the SRMR value was less than 0.08 for all survey years. These results validate the robustness and reliability of the women’s empowerment index across all three NFHS surveys (Supplementary 1).

#### 2.3.3 Background variables

Age of the child in months, Sex of the child, Birth weight, Birth order, Child anemia status, Age of the mother, Delivery by C-section, Institutional delivery, ANC visits, Mother’s Body Mass index, Contraceptive use, Mother’s anemia status, Mother’s occupation, Wealth quintile, Caste, Religion, Place of residence, and Region.

### 2.4 Statistical analysis

We have used descriptive analysis and binary logistic regression to decode the relationship between child undernutrition and women’s empowerment. Binary logistic regression is used to understand the predictors of child undernutrition in India. Before running the regression analysis, all the assumptions have been checked, and there was no multicollinearity (VIF < 3). The basic form of the logistic regression model, which yields the probability of occurring of an event, can be depicted as:


$${\text{log}}_{e}[P(Y_{i}=1|X_{i})/1-P(Y_{i}=1|X_{i})]={\text{log}}_{e}[\pi |1-\pi ]=\alpha +\beta_{1}X_{i1},\ldots \ldots \ldots .\beta_{k}X_{ik}.$$

Where *Y_i_* is the binary response variable, *X_i_* is the set of explanatory variables, and β1, β2…… βk are the coefficients of the *X_i_* variables.

To assess the disparity from 2016 to 2021, we have used multivariate decomposition analysis to see the factors affecting and contributing to the reduction of undernutrition among children. Powers (30) suggested that nonlinear response outcomes be tested to determine the time-period differences in child undernutrition (30). The decomposition analysis was carried out by considering the 2015–16 and 2019–21 outcome groups, respectively. The overall difference in a measured outcome can be decomposed into a sum of components owing to group differences in risk factors and group differences in the effects of those characteristics (30). Specifically, the difference in overall rates for two groups, labelled A and B can be decomposed as


$$r_{A}-r_{B}=F(X_{A}\beta_{A}-X_{B}\beta_{B})$$

(a) $$\underset{E}{\underset{︸}{=r_{A}-r_{B}=[F\bar{\left(X_{A}\beta_{A}\right)}-F\bar{\left(X_{B}\beta_{A}\right)}]\;}}+\underset{C}{\underset{︸}{\;[F\bar{\left(X_{B}\beta_{A}\right)}-F\bar{\left(X_{B}\beta_{A}\right)}]\;}}$$

Where r denotes the child undernutrition in each population and F(X*β*) denotes a once differentiable function mapping a linear combination of risk factors X and effects *β*, in the below multivariate model


(b) $$r=F(X_{g}\beta_{g}),g\in \{A,B)$$

In the above equation (b), r denotes the N × 1 vectors of rates, X is a N × K matrix of independent variables, and β is a K × 1 vector of logistic regression coefficients. The results of the multivariate model are estimated separately. Here we have chosen the reference group to be 2015–16, (the group labelled B) and the comparison group to be 2019–21 labelled as A. The multivariate decomposition splits the child undernutrition difference into two components-endowment (E) and coefficient (C) in equation (a). The “endowment” is the part that can be attributed to the change in the composition of a set of indicators. The “coefficient” is the portion that can be attributed to the change in the effect of indicators included in the analysis. This decomposition approach addresses important questions concerning the potential impacts of equalizing characteristics across the group.

This study has been analyzed on STATA Version 17 All the results were derived by applying the sampling weight provided by the Demographic and Health Survey (DHS) India.

## 3 Results

Table 1 shows the prevalence of undernutrition among children under age five in India from 2006 to 2021. The overall prevalence of undernutrition among children is 42.9% in NFHS-3, 35.9% in NFHS-4, and 31.8% in NFHS-5. Undernutrition prevalence declines over the years, yet the rate remain high among older children, particularly for 46–59 months (NFHS-3 = 45.9%; NFHS-4 = 38.5%; NFHS-5 = 34.2%). Female children exhibit lower undernutrition rates than males across all survey rounds. Children with higher birth order and low birth weight persistently have high undernutrition rates across all NFHS rounds.

**Table 1:** Prevalence of undernourished children under age five by the background variables, NFHS 3, 4, and 5, India.

Background variables	NFHS 3			NFHS 4			NFHS 5		
	Frequency	Percent	Total	Frequency	Percent	Total	Frequency	Percent	Total
Child’s age									
0–6 months	860	30.9	2,785	1,002	25.9	3,861	1,004	28.5	3,528
7–11 months	1,223	35.6	3,435	893	26.7	3,351	677	25.8	2,623
12–17 months	1,645	40.1	4,107	1,288	32.7	3,939	964	30	3,215
18–23 months	1,851	45.4	4,080	1,530	37.9	4,034	985	31.7	3,110
24–34 months	3,330	44.2	7,533	2,566	36.3	7,076	1,949	33.3	5,844
35–45 months	3,460	45.2	7,661	2,796	37.4	7,487	1,903	32.7	5,824
46–59 months	4,430	45.9	9,642	3,431	38.5	8,908	2,596	34.2	7,593
Child’s sex									
Female	8,084	43.3	18,686	6,526	34.6	18,847	4,676	30.6	15,265
Male	8,716	42.4	20,557	6,980	35.2	19,807	5,403	32.8	16,472
Birth order									
One	4,191	35.9	11,670	4,583	30.7	14,916	3,493	27.9	12,531
Two to three	7,232	41.6	17,397	6,480	35.2	18,390	5,079	32.9	15,442
Four and above	5,377	52.8	10,176	2,443	45.7	5,348	1,506	40	3,764
Birth weight									
Low	1,559	45.4	3,431	2,280	45.4	5,018	2,159	41.8	5,171
Normal and above	3,550	27.6	12,881	7,327	29.7	24,677	6,774	28.7	23,623
Child’s anemia status									
Non-anemic	3,643	35.3	10,331	4,477	31	14,445	2,462	27.7	8,899
Anemic	11,072	47.5	23,292	7,927	39.6	20,037	6,425	34.5	18,649
Mother’s age									
15–24	6,712	41.7	16,088	4,579	34.6	13,251	3,337	31.5	10,580
25–34	8,416	42.5	19,799	7,660	34.5	22,228	5,918	31.8	18,627
35 and above	1,672	49.8	3,356	1,267	39.9	3,176	823	32.5	2,531
Delivery by C-section									
No	15,974	44.6	35,852	11,877	37.7	31,541	8,407	33.8	24,900
Yes	810	24.1	3,357	1,629	22.9	7,113	1,671	24.4	6,837
Institutional delivery									
No	11,934	49.8	23,975	3,405	45.2	7,531	1,425	40.1	3,552
Yes	4,863	31.9	15,255	10,102	32.5	31,123	8,653	30.7	28,185
ANC visit									
Less than four	8,353	48.7	17,155	5,122	39.3	13,026	3,331	34.4	9,697
Four or more	2,842	30.4	9,335	4,413	28.9	15,259	4,078	28.5	14,298
Women BMI									
Underweight	7,471	51.6	14,473	4,285	48.1	8,916	2,537	41.9	6,049
Normal	6,397	39.4	16,251	5,670	34.2	16,589	4,650	32.9	14,133
Overweight/Obese	1,110	22.6	4,911	2,139	21.8	9,823	2,107	22.8	9,255
Anemia status of mother									
Non-anemic	5,668	38.9	14,562	5,211	32.4	16,081	3,789	29.8	12,728
Anemic	10,123	45.4	22,309	8,215	36.9	22,254	6,179	33.3	18,560
Contraceptive use									
No	9,692	46.9	20,676	7,879	36.7	21,444	3,984	32.3	12,329
Yes	7,108	38.3	18,567	5,627	32.7	17,210	6,094	31.4	19,408
Occupation of the mother									
Not in the work force	9,462	38.5	24,562	10,141	33.60	30,164	7,854	31.4	25,023
Agricultural	5,338	52.9	10,099	2,013	43.80	4,594	1,261	37.7	3,348
Other	1,990	43.7	4,555	1,237	34.30	3,606	963	28.6	3,366
Women’s empowerment									
Low	6,226	48.4	12,862	5,632	43.8	12,845	3,763	35.6	10,571
Medium	5,836	44.5	13,112	4,603	35.8	12,852	3,453	32.9	10,504
High	4,738	35.7	13,269	3,271	25.2	12,958	2,862	26.8	10,662
Wealth quintile									
Poorest	5,566	57.5	9,672	4,544	49.5	9,186	3,354	43.6	7,699
Poorer	4,331	49.8	8,693	3,407	40.6	8,384	2,415	34.3	7,035
Middle	3,316	41.9	7,921	2,564	32.3	7,930	1,923	30.6	6,294
Richer	2,463	33.8	7,290	1,815	25.9	7,011	1,460	25.2	5,794
Richest	1,124	19.8	5,666	1,175	19.1	6,143	926	18.9	4,914
Community									
SC/ST	5,844	50.6	11,547	4,826	41.3	11,697	3,800	36	10,556
OBC	6,838	43.5	15,714	6,109	35.2	17,365	4,373	31.4	13,945
Others	3,547	34	10,444	2,107	26.7	7,906	1,454	25.3	5,738
Religion									
Hindu	13,464	43.6	30,858	10,886	35.8	30,409	8,089	32.3	25,046
Muslim	2,734	41.9	6,532	2,110	32.9	6,424	1,658	31.4	5,289
Others	602	32.5	1,853	510	28	1,821	331	23.6	1,402
Place of residence									
Urban	3,209	32.9	9,764	3,079	27.8	11,085	2,133	26.2	8,153
Rural	13,591	46.1	29,479	10,427	37.8	27,569	7,946	33.7	23,584
Region									
North	2,288	57.5	3,982	1,462	28.7	5,095	1,072	24.1	4,444
Central	5,614	49.4	11,355	4,108	41.4	9,929	2,708	31.8	8,519
East	2,070	39.1	5,664	3,710	37.3	9,492	3,185	36.6	8,528
Northeast	553	35.2	1,569	340	28.4	1,300	336	26.1	1,184
West	2,081	32	6,502	1,921	35.9	5,357	1,411	36.7	3,848
South	2,075	40.2	5,168	1,965	26.3	7,480	1,368	25.2	5,214
Total	14,682	42.9	34,239	13,506	34.9	38,654	10,078	31.8	31,737

Anemia affects child nutrition significantly, there has been a consistently high prevalence of undernutrition among children if children themselves or their mother have higher rate of anemia. Further, Children of young mothers, particularly those aged 15–24, have higher undernutrition rates. Non-C-section deliveries show higher undernutrition rates. Whereas, children whose mother visited four or more ANC have lower prevalence of undernutrition.

Children of highly empowered mothers consistently show lower undernutrition rates. This trend is evident across all survey rounds, additionally, from NFHS-3 (37.5%) to NFHS-5 (26.8%), there is a 10 percent decline in the prevalence of child undernutrition among highly empowered women.

Socioeconomic and demographic disparities persist. Children from poorer households, SC/ST communities, rural areas, and regions like North and Central India exhibit higher undernutrition rates.

Tables 2–4 present binary logistic regression estimates on child undernutrition determinants, with a specific emphasis on the pivotal role of women’s empowerment.

**Table 2:** Estimates of binary logistic regression of undernourished children under age five by background variables and women’s empowerment, NFHS 3, India.

Background variables	Model 1		Model 2		Model 3		Model 4		Model 5	
	Coef.	95% CI	Coef.	95% CI	Coef.	95% CI	Coef.	95% CI	Coef.	95% CI
Mother’s empowerment index										
Low ^a^										
Middle	−0.16***	[−0.20, −0.11]	−0.17**	[−0.27, −0.07]	−0.08*	[−0.14, −0.01]	−0.01	[−0.07,0.04]	0.0001	[−0.08,0.08]
High	−0.52***	[−0.57, −0.48]	−0.48***	[−0.58, −0.39]	−0.20***	[−0.27, −0.13]	−0.09**	[−0.15, −0.03]	−0.01	[−0.10,0.08]
Child’s age										
0–6 months ^a^										
7–11 months			0.15	[−0.24,0.55]					0.46***	[0.19,0.73]
12–17 months			0.45*	[0.06,0.84]					0.66***	[0.39,0.93]
18–23 months			0.53**	[0.14,0.92]					0.92***	[0.65,1.18]
24–34 months			0.70***	[0.32,1.08]					1.01***	[0.74,1.27]
35–45 months			0.76***	[0.38,1.14]					1.08***	[0.81,1.35]
46–59 months			0.91***	[0.53,1.29]					1.03***	[0.76,1.30]
Child’s sex										
Female ^a^										
Male			−0.06	[−0.13,0.02]					0.01	[−0.05,0.08]
Birth order										
One ^a^										
Two to three			0.13**	[0.05,0.21]					0.19***	[0.10,0.28]
Four and above			0.59***	[0.46,0.71]					0.30***	[0.18,0.42]
Birth weight										
Low ^a^										
Normal and above			−0.76***	[−0.85, −0.67]					−0.53***	[−0.60, −0.45]
Child’s anemia status										
Non-anemic^a^										
Anemic			0.42***	[0.34,0.50]					0.36***	[0.29,0.44]
Mother’s age										
15–24^a^										
25–34					0.22***	[0.16,0.28]			0.02	[−0.06,0.10]
35 and above					0.46***	[0.37,0.55]			0.20**	[0.06,0.34]
Delivery by C-section										
No^a^										
Yes					−0.26***	[−0.36, −0.15]			−0.14*	[−0.26, −0.01]
Institutional delivery										
No^a^										
Yes					−0.28***	[−0.34, −0.21]			−0.11**	[−0.20, −0.03]
ANC visit										
less than 4^a^										
Four or more					−0.32***	[−0.39, −0.26]			−0.13**	[−0.21, −0.05]
Modern contraceptive use										
No^a^										
Yes					−0.09***	[−0.14, −0.04]			−0.22***	[−0.30, −0.15]
Mother’s BMI										
Underweight^a^										
Normal					0.33***	[0.27,0.40]			−0.40***	[−0.47, −0.33]
Overweight					0.21***	[0.13,0.29]			−0.72***	[−0.84, −0.60]
Mother’s anemia status										
Non-anemic^a^										
Anemic					0.12***	[0.07,0.17]			0.06	[−0.01,0.13]
Occupation of mother										
Not working^a^										
Agricultural					0.33***	[0.27,0.40]			0.15***	[0.07,0.23]
Other					0.21***	[0.13,0.29]			0.11*	[0.01,0.21]
Wealth quintile										
Poorest^a^										
Poorer							−0.21***	[−0.28, −0.15]	−0.11*	[−0.21, −0.02]
Middle							−0.51***	[−0.58, −0.44]	−0.33***	[−0.43, −0.22]
Richer							−0.85***	[−0.93, −0.78]	−0.55***	[−0.66, −0.43]
Richest							−1.48***	[−1.58, −1.38]	−0.92***	[−1.07, −0.77]
Caste										
SC/ST^a^										
OBC							−0.12***	[−0.18,−0.07]	-0.07	[−0.15,0.01]
others							−0.35***	[−0.41, −0.28]	−0.29***	[−0.38, −0.19]
Religion										
Hindu^a^										
Muslim							0.12***	[0.05,0.19]	0.1	[−0.01,0.20]
Others							−0.17**	[−0.27, −0.06]	−0.04	[−0.20,0.11]
Place of residence										
Urban^a^										
Rural							−0.05	[−0.11,0.01]	−0.14**	[−0.23, −0.05]
Region										
North^a^										
Central							0.46***	[0.37,0.55]	0.40***	[0.28,0.53]
East							0.20***	[0.13,0.27]	0.22***	[0.12,0.32]
Northeast							−0.39***	[−0.53, −0.26]	−0.42***	[−0.61, −0.22]
West							0.22***	[0.13,0.30]	0.16**	[0.04,0.28]
South							−0.26***	[−0.34,−0.18]	-0.1	[−0.22,0.02]

Coef., Regression Coefficient; CI, Confidence Interval; *p < 0.05, **p < 0.01, ***p < 0.001. ^a^ Reference category.

**Table 3:** Estimates of binary logistic regression of undernourished children under age five by background variables and women’s empowerment, NFHS 4, India.

Background variables	Model 1		Model 2		Model 3		Model 4		Model 5	
	Coef.	95% CI	Coef.	95% CI	Coef.	95% CI	Coef.	95% CI	Coef.	95% CI
Mother’s empowerment index										
Low^a^										
Middle	−0.25***	[−0.31, −0.20]	−0.22***	[−0.28, −0.15]	−0.12***	[−0.19, −0.05]	−0.12***	[−0.18, −0.07]	−0.11*	[−0.20, −0.02]
High	−0.40***	[−0.46, −0.35]	−0.33***	[−0.40, −0.27]	−0.19***	[−0.26, −0.12]	−0.13***	[−0.19, −0.07]	−0.12**	[−0.21, −0.03]
Child’s age										
0–6 months^a^										
7–11 months			−0.15	[−0.41,0.11]					−0.17	[−0.45,0.11]
12–17 months			0.14	[−0.11,0.40]					0.12	[−0.16,0.39]
18–23 months			0.37**	[0.12,0.62]					0.45**	[0.17,0.72]
24–34 months			0.35**	[0.10,0.60]					0.45**	[0.17,0.72]
35–45 months			0.45***	[0.20,0.70]					0.50***	[0.22,0.78]
46–59 months			0.50***	[0.25,0.75]					0.58***	[0.30,0.86]
Child’s sex										
Female^a^										
Male			0.04	[−0.01,0.10]					0.09**	[0.02,0.15]
Birth order										
One^a^										
Two to three			0.20***	[0.14,0.25]					0.19***	[0.10,0.28]
4 and above			0.62***	[0.53,0.71]					0.35***	[0.22,0.49]
Birth weight										
Low^a^										
Normal and above			−0.60***	[−0.66, −0.53]					−0.63***	[−0.72, −0.54]
Child’s anemia status										
Non-anemic^a^										
Anemic			0.34***	[0.29,0.40]					0.21***	[0.14,0.29]
Mother’s age										
15–24^a^										
25–34					0.12***	[0.06,0.19]			−0.04	[−0.13,0.05]
35 and above					0.35***	[0.24,0.45]			0.01	[−0.14,0.16]
Delivery by C-section										
No^a^										
Yes					−0.37***	[−0.45, −0.29]			−0.17***	[−0.26, −0.07]
Institutional delivery										
No^a^										
Yes					−0.18***	[−0.26, −0.11]			0.11	[−0.03,0.25]
ANC visit										
Less than 4^a^										
Four or more					−0.19***	[−0.25, −0.13]			−0.01	[−0.08,0.06]
Modern contraceptive use										
No^a^										
Yes					−0.04	[−0.10,0.02]			−0.14***	[−0.21, −0.06]
Mother’s BMI										
Underweight^a^										
Normal					−0.50***	[−0.56,−0.43]			-0.43***	[−0.51, −0.35]
Overweight					−1.03***	[−1.11, −0.95]			−0.82***	[−0.92, −0.71]
Mother’s anemia status										
Non-anemic^a^										
Anemic					0.10***	[0.05,0.16]			0.07*	[0.00,0.14]
Occupation of mother										
Not working^a^										
Agricultural					0.29***	[0.20,0.37]			0	[−0.11,0.11]
Other					0.11*	[0.02,0.20]			0.09	[−0.02,0.21]
Wealth quintile										
Poorest^a^										
Poorer							−0.30***	[−0.36, −0.23]	−0.27***	[−0.38, −0.16]
Middle							−0.64***	[−0.71, −0.56]	−0.50***	[−0.62, −0.38]
Richer							−0.95***	[−1.04, −0.87]	−0.78***	[−0.92, −0.65]
Richest							−1.34***	[−1.44, −1.24]	−0.97***	[−1.12, −0.81]
Caste										
SC/ST^a^										
OBC							−0.09**	[−0.14, −0.04]	−0.07	[−0.15,0.02]
Others							−0.37***	[−0.44, −0.30]	−0.24***	[−0.35, −0.13]
Religion										
Hindu^a^										
Muslim							0.05	[−0.01,0.12]	−0.05	[−0.16,0.06]
Others							−0.07	[−0.18,0.05]	−0.11	[−0.28,0.07]
Place of residence										
Urban^a^										
Rural							−0.09**	[−0.15, −0.02]	−0.11*	[−0.20,−0.02]
Region										
North^a^										
Central							0.20***	[0.12,0.28]	0.26***	[0.13,0.39]
East							-0.02	[−0.11,0.06]	−0.05	[−0.18,0.08]
Northeast							−0.61***	[−0.78, −0.45]	−0.39**	[−0.64, −0.15]
West							0.31***	[0.22,0.40]	0.25***	[0.11,0.38]
South							−0.17***	[−0.26, −0.09]	0.0500	[−0.13,0.14]

Coef., Regression Coefficient; CI, Confidence Interval; *p < 0.05, **p < 0.01, ***p < 0.001. ^a^ Reference category.

**Table 4:** Estimates of binary logistic regression of undernourished children under age five by background variables and women’s empowerment, NFHS 5, India.

Background variables	Model 1		Model 2		Model 3		Model 4		Model 5	
	Coef.	95% CI	Coef.	95% CI	Coef.	95% CI	Coef.	95% CI	Coef.	95% CI
Mother’s empowerment index										
Low^a^										
Middle	−0.14***	[−0.19, −0.08]	−0.12***	[−0.19, −0.06]	−0.13***	[−0.20, −0.05]	−0.08**	[−0.15, −0.02]	−0.11*	[−0.20, −0.02]
High	−0.43***	[−0.49, −0.37]	−0.33***	[−0.40, −0.26]	−0.32***	[−0.39,−0.24]	-0.2***	[−0.27, −0.14]	−0.15***	[−0.24, −0.06]
Child’s age										
0–6 months^a^										
7–11 months			−0.44**	[−0.73, −0.14]					−0.37*	[−0.69, −0.05]
12–17 months			−0.2	[−0.49,0.08]					−0.14	[−0.45,0.18]
18–23 months			−0.14	[−0.43,0.15]					−0.04	[−0.36,0.27]
24–34 months			−0.01	[−0.29,0.28]					0.1	[−0.21,0.41]
35–45 months			−0.03	[−0.31,0.26]					0.06	[−0.26,0.37]
46–59 months			0.07	[−0.22,0.35]					0.11	[−0.21,0.42]
Child’s sex										
Female^a^										
Male			0.13***	[0.07,0.18]					0.22***	[0.15,0.29]
Birth order										
One^a^										
Two to three			0.26***	[0.20,0.32]					0.28***	[0.19,0.37]
Four and above			0.49***	[0.40,0.58]					0.36***	[0.22,0.50]
Birth weight										
Low^a^										
Normal and above			−0.57***	[−0.64, −0.50]					−0.57***	[−0.66, −0.48]
Child’s anemia status										
Non-anemic^a^										
Anemic			0.36***	[0.30,0.42]					0.26***	[0.18,0.34]
Mother’s age										
15–24^a^										
25–34					0.12***	[0.06,0.19]			0.07	[−0.02,0.17]
35 and above					0.15*	[0.03,0.26]			−0.09	[−0.24,0.06]
Delivery by C-section										
No^a^										
Yes					−0.24***	[−0.31, −0.16]			−0.08	[−0.17,0.01]
Institutional delivery										
No^a^										
Yes					−0.19***	[−0.30, −0.09]			−0.04	[−0.19,0.12]
ANC visit										
Less than 4^a^										
Four or more					−0.19***	[−0.25, −0.13]			−0.10*	[−0.17, −0.02]
Modern contraceptive use										
No^a^										
Yes					0.07*	[0.00,0.14]			0.01	[−0.07,0.09]
Mother’s BMI										
Underweight^a^										
Normal					−0.36***	[−0.44, −0.28]			−0.27***	[−0.36, −0.18]
Overweight					−0.74***	[−0.83, −0.65]			−0.62***	[−0.73, −0.52]
Mother’s anemia status										
Non-anemic^a^										
Anemic					0.07*	[0.01,0.14]			−0.02	[−0.09,0.06]
Occupation of mother										
Not in the work force^a^										
Agricultural					0.18***	[0.08,0.27]			0.05	[−0.06,0.16]
Other					−0.08	[−0.18,0.02]			−0.1	[−0.21,0.01]
Wealth quintile										
Poorest^a^										
Poorer							−0.36***	[−0.43, −0.28]	−0.22***	[−0.33, −0.11]
Middle							−0.51***	[−0.59, −0.42]	−0.28***	[−0.40, −0.16]
Richer							−0.78***	[−0.87, −0.69]	−0.42***	[−0.55, −0.29]
Richest							−1.06***	[−1.17, −0.95]	−0.69***	[−0.84, −0.53]
Caste										
SC/ST^a^										
OBC							−0.06*	[−0.12, −0.00]	−0.10*	[−0.19, −0.02]
Others							−0.29***	[−0.37, −0.21]	−0.29***	[−0.41, −0.18]
Religion										
Hindu^a^										
Muslim							0.04	[−0.03,0.12]	0.16**	[0.05,0.27]
Others							−0.28***	[−0.42, −0.14]	−0.27**	[−0.46, −0.09]
Place of residence										
Urban^a^										
Rural							−0.03	[−0.10,0.04]	−0.03	[−0.12,0.06]
Region										
North^a^										
Central							0.11*	[0.02,0.20]	0.24***	[0.12,0.37]
East							0.29***	[0.20,0.39]	0.41***	[0.27,0.54]
Northeast							−0.16	[−0.34,0.02]	0.09	[−0.15,0.32]
West							0.54***	[0.43,0.64]	0.58***	[0.44,0.72]
South							0.06	[−0.04,0.16]	0.26	[0.12,0.40]

Coef., Regression Coefficient; CI, Confidence Interval; *p < 0.05, **p < 0.01, ***p < 0.001. ^a^ Reference category.

Model 1 shows the unadjusted results from all three surveys, which consistently revealed a significant negative association between higher levels of women’s empowerment and child undernutrition. Upon adjustment for child-specific factors in Model 2, the significance of women’s empowerment on child undernutrition persists across all surveys. However, the effect was stronger in NFHS-3, where children with more empowered mothers were 48 % less likely to experience undernutrition.

Model 3, focusing solely on maternal variables, further reinforces the significant role of women empowerment in reducing child undernutrition. After controlling maternal characteristics such as age, delivery method, ANC visits, anemia status, occupation and women’s empowerment, higher levels of women’s empowerment continue to show a consistent negative association with child undernutrition in all three surveys. Model 4 (adjusted by all the household variables) demonstrates that despite the mediating influence of household factors, women’s empowerment remains a significant predictor of child undernutrition status in NFHS 3, 4, and 5. However, the effect and strength of the association were weakened in NFHS-3 (coef = −0.09**; CI [−0.15, −0.03]).

Further, the completely adjusted model (Model 5) indicate the effect of women’s empowerment on their child undernutrition when all covariates are considered. In NFHS-3 (2005–06), the impact of women’s empowerment was mitigated entirely in the presence of all the considered covariates. However, in NFHS-4, empowerment emerges as one of the crucial drivers of child undernutrition (coef = −0.12**; CI [−0.21, −0.03]), and by NFHS-5, this association and magnitude become more strong (coef = −0.15***; CI [−0.24, −0.06]).

The age of the child exhibits a significant negative association with undernutrition status in NFHS-3 and NFHS-4, indicating that as the child ages, the risk of undernutrition increases. Whereas, in NFHS-5 the effect of age was completely mitigated. Birth order, birth weight, and anemia status of the children emerge as significant contributors across all surveys, with higher birth order or child anemia increasing the risk of undernutrition, while normal or higher birth weight reduces this risk.

In NFHS-3, maternal age, institutional delivery, cesarean delivery, ANC visits, and maternal anemia are identified as significant Drivers to the risk of undernutrition among children. However, over time, the influence of these variables appears to diminish, suggesting potential shifts in maternal and healthcare practices or broader socioeconomic changes affecting child nutritional outcomes.

Further, the mother’s BMI stands out as a significant determinant of her child’s nutritional status across the years from 2006 to 2021. Additionally, the household’s wealth status emerges as a prominent driver of child undernutrition under the age of five in all the survey years.

Table 5 provides a broad perspective into the complex interplay of inherent characteristics (Endowment) and changes in influential factors (Coefficient). The multivariate decomposition analysis reveals that 30.35% of the reduction in child undernutrition between NFHS-4 and NFHS-5 is attributable to changes in endowments (due to differences in characteristics), while 69.65% is due to changes in coefficients (due to difference in coefficients). The total predicted change in undernutrition is 2.7 percentage points.

**Table 5:** Multivariate decomposition of undernourished child under age five with background and women’s empowerment variables, NFHS 4 to NFHS 5, India.

NFHS 4 to NFHS 5					
Background variables	Coefficient	P > z	95% confidence interval		Percent contribution
			Lower limit	Upper Limit	
Endowment	0.008	0.000	0.005	0.012	30.35
Coefficient	0.019	0.000	0.009	0.029	69.65
R	0.027	0.000	0.017	0.037	
Due to differences in characteristics					
Child’s age	−0.0012	0.001	−0.0019	−0.0005	−4.38
Child’s sex	−0.0003	0.047	−0.0007	0.0001	−1.25
Birth order	−0.0014	0.007	−0.0024	−0.0004	−5.24
Birth weight	0.0070	0.001	0.0027	0.0114	26.03
Child’s anemia status	0.0019	0.000	0.0009	0.0029	6.88
Mother’s age	0.0000	0.835	−0.0003	0.0004	0.13
Delivery C-section	−0.0001	0.021	−0.0001	0.0000	−0.27
Institutional delivery	−0.0003	0.183	−0.0008	0.0002	−1.16
ANC visits	0.0000	0.902	−0.0001	0.0001	0.03
Mother’s BMI	0.0017	0.002	0.0006	0.0028	6.37
Mother’s anemia status	−0.0001	0.136	−0.0002	0.0000	−0.26
Use of modern contraception	−0.0020	0.043	−0.0040	−0.0001	−7.46
Women’s empowerment	0.0009	0.022	0.0001	0.0017	3.35
Wealth quintile	0.0063	0.001	0.0026	0.0101	23.41
Caste	0.0018	0.005	0.0005	0.0030	6.53
Religion	0.0006	0.051	0.0000	0.0011	2.04
Place of residence	0.0005	0.045	0.0000	0.0009	1.74
Region	0.0002	0.624	−0.0007	0.0012	0.86
Due to difference in coefficients					
Child’s age	0.06265	0.004	0.0206	0.10476	231.85
Child’s sex	−0.01621	0.023	−0.03016	−0.00226	−60
Birth order	−0.0076	0.722	−0.04946	0.03425	−28.13
Birth weight	−0.04677	0.129	−0.10717	0.01363	−173.08
Child’s anemia status	−0.00509	0.598	−0.02401	0.01383	−18.84
Mother’s age	0.01445	0.531	−0.03075	0.05966	53.48
Delivery C-section	−0.00454	0.288	−0.01293	0.00385	−16.82
Institutional delivery	0.03647	0.157	−0.01409	0.08703	134.97
ANC visits	0.01606	0.070	−0.00133	0.03346	59.44
Mother’s BMI	−0.05422	0.016	−0.09834	−0.01011	−200.67
Mother’s anemia status	0.01068	0.165	−0.00438	0.02575	39.53
Use of modern contraception	−0.03901	0.001	−0.06126	−0.01675	−144.35
Women’s empowerment	0.00284	0.867	−0.03033	0.03601	10.52
Wealth quintile	−0.03545	0.062	−0.07274	0.00184	−131.2
Caste	−0.00736	0.672	−0.04147	0.02675	−27.25
Religion	−0.01299	0.410	−0.04388	0.01791	−48.06
Place of residence	−0.03342	0.237	−0.08881	0.02197	−123.68
Region	−0.05158	0.000	−0.0781	−0.02502	−190.88

Among endowment-based contributors, birth weight emerged as the most influential factor, accounting for 26.03% of the reduction followed by wealth quintile (23.41%), and mother’s BMI (6.37%). Women’s empowerment contributed 3.35%, which, although lower than economic or biological determinants, is notable given its socio-behavioral nature. In contrast, factors such as higher birth order (−5.24%), and older child age (−4.38%) were associated with widening the gap, contributing negatively to the nutritional improvement.

The group differences in the effects of these predictors (due to coefficients) contribute 70 %, of which only the child’s age, Child’s sex, Mother’s BMI and the mother’s contraceptive use are significant contributors.

While women’s empowerment contributed less than traditional determinants like wealth and birth weight, its positive and significant influence underscores the growing relevance of gender-based interventions in public health.

## 4 Discussion

The current study embarks on a thorough exploration spanning three distinct time periods. It detangles the relationship between women’s empowerment and child undernutrition in India, highlighting that focusing on women’s empowerment may help to combat child undernutrition effectively. Existing research predominantly centers on limited dimensions of empowerment, such as women’s autonomy, control, and decision-making power within the household. Although maternal involvement in decision-making generally aligns with improved child nutritional status however, the strength and direction of these connections vary across different sub-domains of decision-making and depend on contextual factors such as child age, household wealth, and social support networks (16). Moreover, the domains of women’s empowerment are interconnected and interdependent. Neglecting certain aspects of empowerment may undermine the effectiveness of interventions aimed at improving child nutrition (15, 16, 31, 32). Women’s empowerment encompasses various dimensions and each of these domains plays a distinct yet interconnected role in shaping maternal and child health outcomes. (33, 34). While women’s empowerment, as measured by participation in household decision-making, freedom of mobility, and ownership, represents a distinct aspect of empowerment. Focusing solely on limited dimensions may overlook the broader context in which women navigate their lives and make choices regarding their own and their children’s health. Hence, the study utilizes women’s empowerment based on six dimensions to study its relationship with their child’s undernutrition.

One of the key finding of our study highlights the susceptibility of male children to undernutrition. Various studies support this finding that male children are more likely to be undernourished than female children, especially in the recent year (2019–21), which aligns with several studies as well (35–37). This disparity is multifaceted and can be attributed to a combination of biological, social, and environmental factors. Biologically, male children may face inherent vulnerabilities that predispose them to undernutrition, such as differences in metabolism, nutrient absorption, and susceptibility to infections. A comprehensive systematic review and meta-analysis of sex differences in undernutrition sheds further light on the complexity of this issue. The analysis revealed that approximately 14 % of the studies identified biological factors as primary contributors to the disparity, highlighting the importance of understanding the physiological differences between male and female children. However, a significant majority (39% of studies) shows that this disparity is due to the combination of social, and environmental factors in shaping the undernutrition landscape among children (37).

Our study examined the effect of birth order and birth weight on child undernutrition and found that a higher birth order and lower birth weight are associated with a higher risk of undernutrition. A study done by Rahman (11) in Bangladesh indicates that there are 38 % of children are stunted, of which children with fifth or higher birth order have approximately 70 % higher likelihood of being stunted (11). Another study done in sub-Saharan Africa found that low birth weight is a key determinant of undernutrition among children under age five years (13). Children with low birth weight are more likely to be underweight and prone to contracting diseases and infections, such as anemia and respiratory infections, which can increase their likelihood of being underweight (13).

Despite the widespread recognition of women’s empowerment as a pivotal factor in improving child nutrition outcomes, our findings present a complex picture. This study shows that women’s empowerment may not directly influence child undernutrition during the years 2005–06. This contrasting result may stem from population-specific factors, contextual variations, and methodological differences in measurement and analysis. Across various studies, a significant divergence exists in how empowerment is conceptualized and assessed (24, 38, 39). This lack of uniformity in methodologies and classification of empowerment indicators frequently results in conflicting conclusions regarding their correlation with child nutritional well-being, which demands a standardized measurement approach to draw definitive conclusions about the impact of women’s empowerment (32). Another possible reason is that key socioeconomic and demographic factors may have diluted the effect of women’s empowerment, as they serve as primary determinants of child nutrition (40). Furthermore, the study offers key insights into the changing dynamics of women’s empowerment on child undernutrition. By 2015–16, women’s empowerment emerged as a key driver of child undernutrition and contributed to a 3.3% reduction in undernutrition from 2015–16 to 2019–21, underscoring the transformative power of women’s empowerment in reshaping societal norms and public health outcomes.

The recognition of women’s empowerment as a critical determinant of child nutrition outcomes is well-supported in literature (15, 31, 32, 40). A systematic review published in 2019 emphasized the pivotal role of women’s empowerment, particularly during the first thousand days of a child’s life, in shaping nutritional outcomes. The review highlighted the need for further exploration of the pathways linking women’s empowerment to child nutrition, emphasizing its importance in public health discourse (32). Additionally, a longitudinal analysis conducted in India validated these findings, highlighting the positive influence of women autonomy on children’s health outcomes (41). Factors such as decision-making authority, freedom of movement, and financial autonomy were identified as key contributors to improved child nutrition in households where women enjoyed higher levels of empowerment.

The shifting landscape of child undernutrition in India reflects broader societal changes driven by various policies and programs aimed at empowering women and enhancing maternal and child health outcomes. Initiatives like the Integrated Child Development Services (ICDS) program, a flagship initiative, recognize the integral role of women in ensuring child health and nutrition (42). The National Rural Health Mission (NRHM), now part of the National Health Mission (NHM), focuses on enhancing healthcare delivery in rural areas, with maternal and child health as a crucial component (43, 44). Additionally, programs like the Janani Suraksha Yojana (JSY) and Pradhan Mantri Matru Vandana Yojana (PMMVY) aim to incentivize institutional deliveries and provide financial assistance to pregnant and lactating women. The National Nutrition Mission, or POSHAN Abhiyaan, launched in 2018, represents a concerted effort to address malnutrition comprehensively (27, 45, 46). By focusing on the first 1,000 days of a child’s life, this mission aims to empower women through targeted interventions, emphasizing the importance of nutrition, health, and sanitation. Moreover, initiatives such as Beti Bachao, Beti Padhao (Save the Girl Child, Educate the Girl Child) spotlight to escalate the education among female children (47). By challenging gender-based discrimination and promoting education for girls, these programs contribute to a broader narrative of empowering women to break the chains of malnutrition.

## 5 Recommendation(s)

Based on the above study, the following recommendations can be used to enhance the effectiveness of the existing program and policy.

1. Enhance Program Synergy and Integration: There is a pressing need to enhance synergy and integration between various programs on women’s empowerment and maternal and child health programs and services. Many initiatives currently operate in silos, which hinders their effectiveness. Integrating these programs would ensure a comprehensive approach to addressing the multifaceted determinants of child nutrition.
2. Targeted Interventions for High-Risk Groups: Modify existing programs to provide more targeted interventions for high-risk groups, such as children from economically disadvantaged households, those with multiple siblings, and regions with a high prevalence of undernutrition.
3. Comprehensive approach toward Women’s empowerment: Programs and policies should promote women’s empowerment across all the domains equally. Strengthening this area creates a supportive environment, enabling women to make better health choices and access essential services. This holistic approach may lead to more sustainable improvements in maternal and child health outcomes.

## 6 Conclusion

This study provides a comprehensive understanding of how women’s empowerment influences child undernutrition in India from 2006 to 2021. It underscores that in 2005–06, empowerment was not a significant factor in reducing child undernutrition, as child, maternal and socioeconomic factors played predominant roles. However, after socioeconomic improvements and interventions in mother–child-related programs and policies, both health and socioeconomic status improved, and empowerment emerged as a significant factor by 2015–16. This shift in the dynamics of child undernutrition aligns with the visionary perspective articulated by Kofi Annan, echoing, “There is no tool for development more effective than empowering women.” As India continues to progress toward achieving its developmental goals, the empowerment of women emerges as a cornerstone in fostering healthier and more prosperous communities, with benefits extending to future generations.

## Data Availability

Publicly available datasets were analyzed in this study. This data can be found at: https://dhsprogram.com/Data/.
