# Supplementary material for: The evolving nexus of women’s empowerment and child nutrition in India
Source: Front Public Health. 2025 Aug 26;13:1583678. doi: 10.3389/fpubh.2025.1583678 (PMC12419225; doi:10.3389/fpubh.2025.1583678)
Supplement: Supplementary file 1 [file Table_1.docx]

Supplementary Material

# Supplementary Tables

Supplementary 1: Description of the domain and variables of women’s empowerment

| **Domain** | **Variables** | **Score** |  |
| --- | --- | --- | --- |
| **Attitude towards Violence** | | |  |
|  | Whether beating is justified if the wife goes out without telling the husband | Justified=0, not justified=1 |  |
|  | Whether beating is justified if the wife neglects the children | Justified=0, not justified=1 |  |
|  | Whether beating is justified if the wife argues with the husband | Justified=0, not justified=1 |  |
|  | Whether beating is justified if the wife refuses to have sex with the husband | Justified=0, not justified=1 |  |
|  | Whether beating is justified if the wife burns the food | Justified=0, not justified=1 |  |
| **Decision-Making** | | |  |
|  | Who Decides on the respondent's healthcare? | Decides alone or with partner=1, else =0 |  |
|  | Who Decides on large household purchases? | Decides alone or with partner=1, else =0 |  |
|  | Who Decides on visits to family or relatives? | Decides alone or with partner=1, else =0 |  |
|  | Who Deciding what to do with the money the husband earns? | Decides alone or with partner=1, else =0 |  |
| **Freedom of Mobility** | | |  |
|  | Are you allowed to go to the market? | Allowed to go alone or with someone, else=0 |  |
|  | Are you allowed to go to the health facility? | Allowed to go alone or with someone=1, else=0 |  |
|  | Are you allowed to go to the village? | Allowed to go alone or with someone=1, else=0 |  |
| **Perceived Sexual Rights** | | |  |
|  | Refused to have sex if the wife knows her husband has sex with other women | Yes=1, No=0 |  |
|  | Say no to having sex if the wife does not want to have sexual intercourse. | Yes=1, No=0 |  |
|  | Refused to have sex if the wife knows her husband has a sexually transmitted disease | Yes=1, No=0 |  |
| **Financial Security** | | |  |
|  | Do you have a bank account? | Yes=1, No=0 |  |
|  | Do you own a house? | Yes=1, No=0 |  |
|  | Do you have a mobile phone? | Yes=1, No=0 |  |
|  | Do you own a land? | Yes=1, No=0 |  |
|  | Have you worked in the last 12 months? | Yes=1, No=0 |  |
|  | Do you have any money of your own that you alone can decide how to use? | Yes=1, No=0 |  |
| **Societal Norm** | | |  |
|  | Use Media exposure (read, radio and TV) | Yes=1, No=0 |  |
|  | Women's education | Secondary and higher=1, No education or primary=0 |  |
|  | Age of respondent at cohabitation | 18 and above=1, else=0 |  |
|  | Age of respondent at first birth | 18 and above=1, else=0 |  |
|  | Age difference: woman’s minus husband’s age | Equal or greater=1, else 0 |  |
|  | Education difference: woman’s minus husband’s years of schooling | Equal or greater=1, else 0 |  |

Supplementary 2: Construct validity of the indices obtained from confirmatory factor analysis, 2005-06, 2015-16 and 2019-2021, India

| Survey | RMSEA | CFI | TLI | SRMR |
| --- | --- | --- | --- | --- |
|  |  |  |  |  |
| 2005-06 | 0.018 | 0.994 | 0.992 | 0.015 |
| 2015-16 | 0.010 | 0.989 | 0.995 | 0.010 |
| 2019-21 | 0.008 | 0.995 | 0.994 | 0.007 |
| RMSEA: Root mean squared error of approximation | | | | |
| CFI: Comparative fit index | | |  |  |
| TLI: Tucker–Lewis index | | |  |  |
| SRMR: Standardized root mean squared residual | | | | |
